# Mutation in *SF3B1* gene promotes formation of polyploid giant cells in Leukemia cells

**DOI:** 10.1007/s12032-022-01652-9

**Published:** 2022-04-28

**Authors:** Sanjay Mukherjee, Abdullah Mahmood Ali, Vundavalli V. Murty, Azra Raza

**Affiliations:** 1 Division of Hematology/Oncology, Department of Medicine, Columbia University Irving Medical Center, New York, NY 10032 USA; 2 Department of Pathology and Cell Biology, and Institute for Cancer Genetics, Department of Medicine, Columbia University Irving Medical Center, New York, NY 10032 USA; 3 Herbert Irving Comprehensive Cancer Center, Columbia University, New York, NY 10032 USA; 4 MDS Center, Columbia University Irving Medical Center, 177 Fort Washington Avenue, Milstein Hospital Building, Room 6GN-435, New York, NY 10032 USA

**Keywords:** Polyploid giant cells, Leukemia, SF3B1, Cancer

## Abstract

Giant cells with polyploidy, termed polyploid giant cells, have been observed during normal growth, development, and pathologic states, such as solid cancer progression and resistance to therapy. Functional studies of polyploidal giant cancer cells (PGCC) provided evidence that they arise when normal diploid cells are stressed, show stem cell-like properties, and give rise to tumors. In the present study, we report in K562 leukemia cell line that introduction of the hotspot K700E mutation in the gene *SF3B1* using CRISPR/Cas9 method results in an increased frequency of multinucleated polyploid giant cells resistant to chemotherapeutic agent and serum starvation stress. These giant cells with higher ploidy are distinct from multinucleated megakaryocytes, are proliferative, and are characterized by increased accumulation of mitochondria. PGCC have been previously documented in solid tumors. This is the first report describing PGCCs in a cell line derived from a liquid cancer where increased frequency of PGCCs is linked to a specific genetic event. Since *SF3B1* mutations are predominantly seen in MDS and other hematologic malignancies, our current findings will have significant clinical implications.

**Supplementary Information:**

The online version contains supplementary material available at 10.1007/s12032-022-01652-9.

## Introduction

Giant cells with polyploidy and multiple nuclei have been observed during normal growth and development as well as in pathologic states, such as cancer initiation, progression, and therapy resistance [1–7]. The polyploid giant cells observed in solid tumors were termed as PGCCs (polyploid giant cancer cells), but other terms including poly‐aneuploid cancer cells (PACC) [8], multinucleated giant cells [9], osteoclast-like giant cells [10], and cancer-associated macrophage-like cells (CAML) [11] are also in use describing multinucleated giant-sized cells. Cancers with proven viral etiology are characterized by PGCC [12]. Functional studies of PGCCs provided evidence that these cells arise from normal diploid cells under stress, show stem cell-like properties, and give rise to tumors [7]. PGCCs in solid tumors are well documented but their existence in liquid tumors, such as acute myeloid leukemia (AML) and myelodysplastic syndromes (MDS,) is not well documented.

MDS is a heterogeneous group of hematopoietic stem cell disorders characterized by cytopenias due to ineffective hematopoiesis and an increased propensity for transformation to AML in a third of the patients [13]. Recurrent cytogenetic abnormalities are characteristic of both MDS and AML [14]. In MDS, almost 50% of the patients show an abnormal karyotype recurrently involving chromosomes 3, 5, 7, 8, 11, 12, 17, 20, and Y but polyploidy is rare [15]. Similarly, in AML, be it de novo or secondary (arising from MDS), aneuploidy is common, but polyploidy is rare [16, 17]. Several cases with near tetraploid to tetraploid cells in both MDS and AML patients have been reported using descriptive terms like “giant” and “bizarre” cells, but the significance of these polyploidal giant cells remains unclear [18–21].

Apart from the cytogenetic abnormalities, somatic mutations in over 40 frequently mutated genes with functions in diverse cellular processes such as DNA methylation, chromatin modification, splicing, and transcription are reported in MDS [22]. Along with DNA methylation, RNA splicing genes are the most frequently mutated in MDS [22]. Particularly, mutations in *SF3B1* have been described in up to 85% patients with a subtype of MDS characterized by ring sideroblasts [22–24].

Previously, our group and others have shown that heterozygous point mutations in *SF3B1* affect pre-mRNA splicing, owing to its role in facilitating the interaction of snRNP spliceosomal complex with branch site, resulting in retention of 10 to 25 nucleotides long-intron upstream of 3’ acceptor splice sites in over 1000 genes [25–27]. This widespread defect in pre-mRNA splicing of many genes results in cellular stress. A subset of the affected genes is involved in a variety of cellular functions, including iron metabolism, erythropoiesis, and mitochondrial activities [28–31].

In this study, we report our observations that introduction of the most frequent hotspot mutation in *SF3B1* gene in K562 cells results in an increased frequency of polyploid multinucleated giant cells that are resistant to serum starvation conditions and commonly used chemotherapeutic agent, azacytidine, approved for the treatment of both MDS and AML. We show that PGCCs are distinct from the occasional megakaryocyte observed in K562 cells. We also show, using cell proliferation markers, cytogenetics, and time-lapse videography, that PGCC are capable of cell division. Finally, the mutant cells show an increase in mitochondrial biomass.

These observations provide the first reported evidence of the appearance of PGCC in liquid cancers and may have implications in the initiation and progression of MDS and AML as well as resistance to chemotherapy.

## Materials and methods

### Cells and cell culture method

The wild-type and *SF3B1* K700E mutation bearing K562 cells were described previously [26, 32]. A synonymous change or mutation (c.2098A > G) was introduced using CRISPR/Cas9 genome engineering method. The wild-type and the mutant K562 cells were cultured in IMDM media (Thermo) supplemented with 10% fetal bovine serum (GE system) and 1% Penicillin–streptomycin (Gibco).

### Flow cytometry

Flow cytometry analysis was carried out in Fortessa II (BD Biosciences) using CellQuest software (BD Biosciences, San Jose CA, USA). Scatter plots and cell quantification analysis were carried out using FlowJo software (version 10.1) (BD Biosciences) or FCS Express 7 (De Novo Software).

### Propidium Iodide DNA staining

The genome content of the wild-type and mutant K562 cells was measured using Propidium Iodide (PI). 4 WT and 4 mutant (K700E) K562 clones were used for the assay. A total of 1 million cells for each experiment were fixed in cold 70% ethanol for 1 h. The cells were washed with 1xPBS twice. The supernatant was discarded and the pellet was resuspended in 1 mL of mixture of Ribonuclease A (50 µL from 100 µg/mL stock) and PI (200 µL from 50 µg/mL stock) in 1xPBS. The cells were stained for 20 min at room temperature in dark. The cells were washed with 1xPBS twice, resuspended in 500 uL 1xPBS, and analyzed using Flow cytometry.

### Nuclear and mitochondrial staining

WT and mutant (K700E) K562 cells were washed with 1xPBS twice. The cells were finally resuspended at a density of 1 million/mL in 1xPBS to which Mitotracker Rhodamine 123 solution (Sigma) was added to a concentration of 50 µM from a 25 mM stock. The cells were incubated in dark for 1 h at room temperature. After 1 h, 10 µg/µL Hoechst 3342 (Thermo) solution was added for nuclear staining and CellBrite Fix 555 (Biotium) for plasma membrane staining to the cells and incubated in dark for 10 min at room temperature. The cells were then washed twice with 1xPBS and resuspended in 100 µL of 1xPBS. Out of which 30 µL was loaded on 35-mm glass bottom plates (MatTeK Inc, USA) and observed under confocal microscope.

### Treatment with 5-azacytidine

Three clones each of WT and K700E mutants were seeded in 6-well plate at a concentration of 3 × 10^5^ cells /well. The cells were treated with 25 µM 5-Azacytidine (SellekChem) (stock 10 mM) or DMSO as control for 72 h in IMDM media supplemented with 10% FBS. For IC50 calculation cells were treated with different concentrations of 5-Azacytidine from 0 to 100 µM for 48 h and 72 h. The cytotoxicity was analyzed using AnnexinV /PI staining and measured through Flow Cytometry.

### Microscopy

Confocal microscopy was carried out using Nikon Ti Eclipse-inverted microscope and analyzed using Nikon NIS-AR Elements software (Nikon Inc, USA). High-resolution images were taken using STORM (Stochastic Optical Reconstruction Microscopy) 100x/1.49 objective lens. Light microscopy and imaging were done using Olympus Ix51 microscope attached with Olympus DP72 camera. Images were further analyzed using Fiji Software (https://imagej.net/Fiji).

### CD61 staining

The CD61 expression analysis in WT and mutant cells was carried out using Flow cytometry and microscopy. Three clones each of WT and K700E mutants were seeded in 6-well plate at a density of 3 × 10^5^ cells per well in 3 sets. One set was treated with 25 µM 5-Azacytidine (SellekChem), one set with 5 nM PMA (Sigma), and one set with 300uM CoCl2 (Sigma). All the treatments were done in IMDM media with 10% FBS for 72 h. For CD61 expression using flow cytometry, cells were washed with 1xPBS twice and stained with 5 uL of CD61 (BioLegend) in 500 uL 1xPBS. Staining was carried out for 20 min at room temperature in dark. The CD61 expression was analyzed using Fortessa II (BD Biosciences).

For microscopy, the CD61-stained cells were further stained with Hoechst (Thermo) for 10 min. The stained cells were washed with 1xPBS, mounted on glass slide, and visualized under Olympus microscope at 20X magnification. The images were captured using Olympus DP74 camera attached to the microscope.

### Time-lapse microscopy

The WT and mutant K562 cells were resuspended in StemCell MethoCult (H4034, StemCell, Inc) media at a concentration of 1 × 10^5^ cells/mL. Cells were then seeded in 96-well glass-bottomed plates (Greiner Inc) at a density of 10^4^ cells/well and monitored for time lapsed for 24 h. Images were captured using BioTek Cytation 5 plate reader with BioSpa 8 automated incubator (BioTek) at a frequency of every 30 min and analyzed using Gen 5 (BioTek) and Fiji (Image J) softwares.

### Metaphase and FISH (fluorescent in situ hybridization) analysis

Actively growing WT and mutant K562 cells in duplicate were processed for metaphase preparations using standard methods [33]. Briefly, cells were treated with hypotonic solution, fixed in methanol:acetic acid fixative and slides were prepared. FISH analysis was performed using a tri-color combination of fluorescent-labeled centromere probes for chromosomes 3 (orange), 7 (green), and chromosome 17 (aqua) obtained from Abbott Molecular (IL, USA).

### Ki-67 staining

For Ki-67 immunofluorescence experiments, overnight cultures of WT and K700E cells (`2 × 10^6^) were first washed with 1xPBS and fixed in 4% paraformaldehyde in PBS for 10 min at room temperature. Cells were then gently washed with ice-cold PBS and permeabilized with 0.5% Triton TX-100/PBS on ice for 10 min. Cells were then washed twice with room-temperature PBS and then incubated with Ki-67 antibody (1:200 dilution in 1xPBS-2.5 ug/mL-Biolegend 350,501) for 1 h at room temperature in dark. The cells were then washed with 1xPBS twice at room temperature. After washing, cells were incubated with secondary anti-mouse Rabbit antibody (1:500 in 1xPBS) labeled with Alexa Fluor 488 (Thermo Life Technology) for 30 min at room temperature. The cells were washed twice with 1xPBS. The nuclei were counterstained with Hoechst as described above and plated in 35-mm glass bottom plates at a concentration of 1 × 10^4^ cells/mL. The images were captured using confocal microscope with 40X objective lens.

### Statistical analysis

All the statistical analysis were carried out using GraphPad Prism 6. *P* values were calculated using unpaired Student’s *t* test (Welch’s Corrected) between 3 to 5 WT and mutant (K700E) clones. The difference was found significant at *P* < 0.05.

## Results

### Mutation in *SF3B1* (K700E) gene results in increased frequency of giant cells in K562 cells

**Fig. 1:**
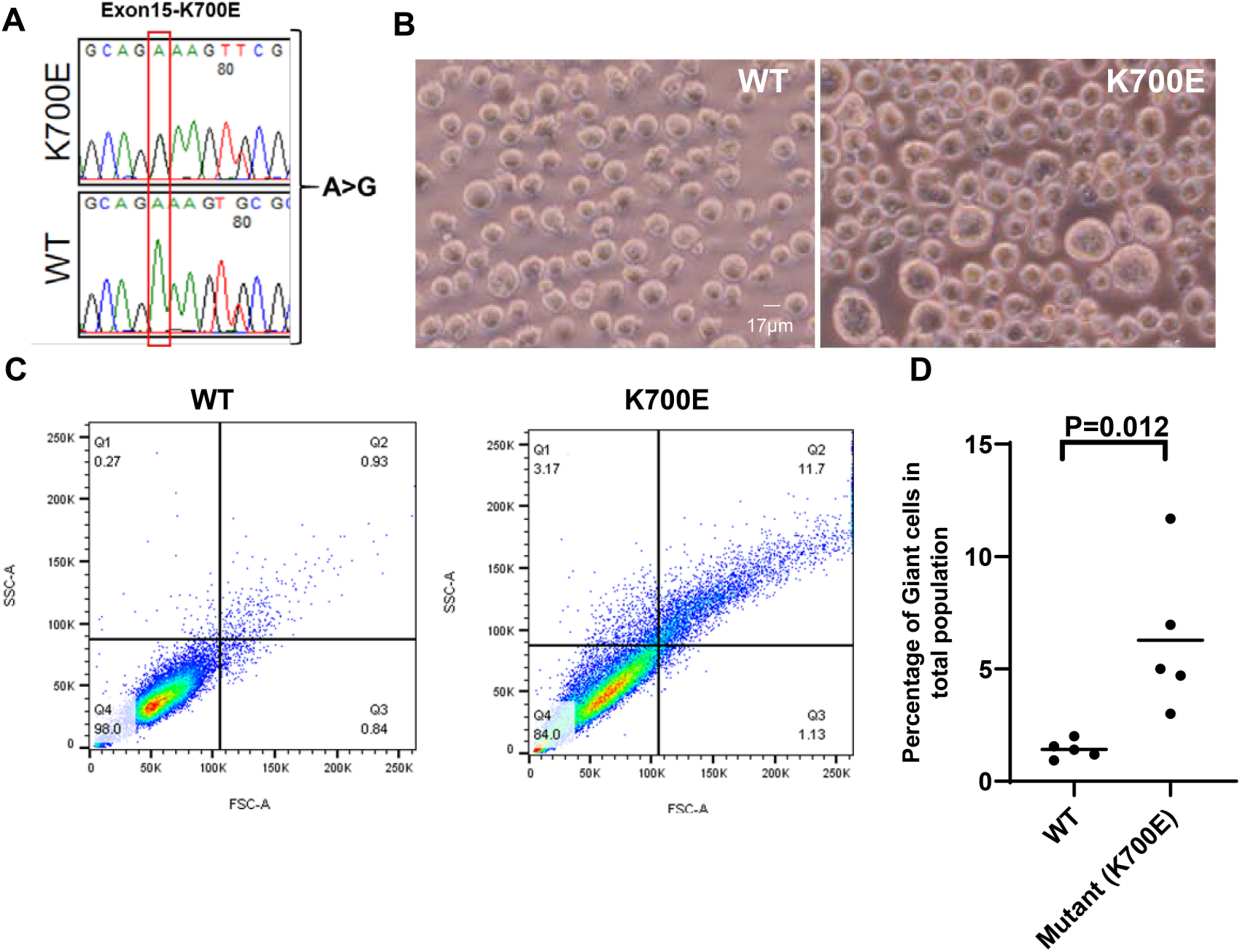
Mutation in *SF3B1* (K700E) gene results in formation of PGCCs in K562 cells. **A** Mutation in the exon 15 of *SF3B1* gene was generated in K562 cells using CRISPR-Cas9. The mutation is ‘A’ to ‘G’ which results in addition of Glutamic acid in place of Lysine at 700 amino acid position in SF3B1. **B** Morphological differences in the wild-type (WT) and mutant (K700E) cells, showing appearance of large-sized cells. **C** A representative scatter plot of one clone each of WT and mutant (K700E) cells. **D** Data from five independent clones are analyzed in each experiment that was repeated three times. Analysis showing six- to tenfold increase (*P* = 0.012) in appearance of giant size cells in mutants compared to WT. *The WT went through same CRISPR-Cas9 procedure without the mutation*

Somatic mutations in *SF3B1* gene are frequently observed in a subset of MDS patients showing characteristic ring sideroblasts [22, 24–26]. To investigate the functional consequences of mutations in *SF3B1*, we used the CRISPR/Cas9 genome engineering method to introduce the hotspot K700E mutation in K562 cells, an erythroleukemia cell line commonly used as a model in MDS and AML [26, 34]. In parallel, we also subjected wild-type K562 cells to the exact same CRISPR/Cas9-mediated genome manipulations except that the wild-type cells had synonymous change [26, 32]. Several independent clones were obtained from both K700E and wild-type (WT) cells (Fig. 1A). In an actively growing culture, we found K700E clones to exhibit higher frequency of giant-sized cells compared to WT (Fig. 1B). The visual difference in size was further documented on flow cytometry forward and side scatter dot plots (Fig. 1C). On average, we noticed a sixfold increase in giant-sized cells in mutant compared to wild-type cells (Fig. 1D). Interestingly, we did not observe increased giant cells in K562 cells when we knocked in another hotspot mutation P95H in the *SRSF2* gene, a splicing factor frequently mutated in MDS (Supplementary Figure S1).

### The giant cells in K700E mutants are polyploid

**Fig. 2:**
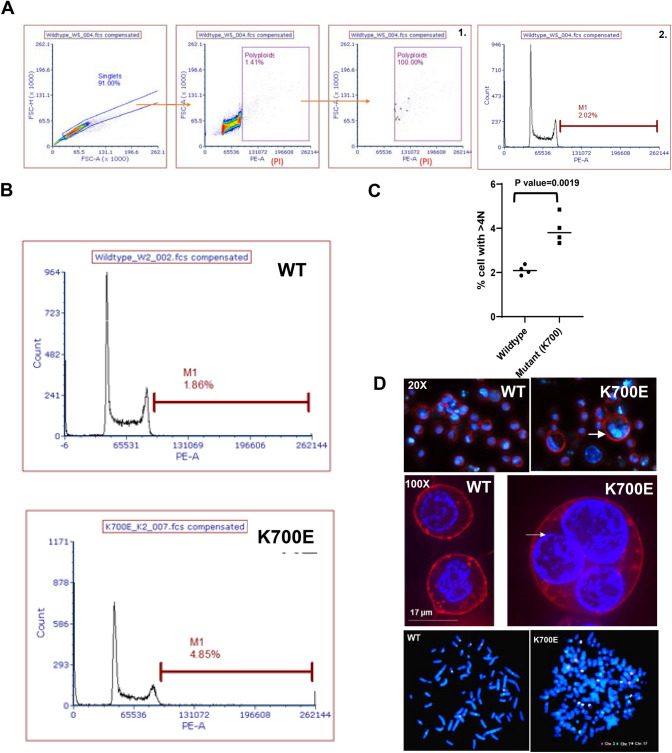
The giant cells in K700E mutants are polyploid. DNA content in WT and mutant cells was analyzed using Propidium iodide (PI)-based cell cycle assay **A** Gating strategy for quantization of polyploid cells by PI-FACS analysis. **B** A representative histogram from a set of four independent clones showing DNA content of WT and K700E mutants. The region marked by brown line indicated by M1 shows count of cells with polyploidy. **C** Mutant cells had significantly higher number of cells with > 4 N DNA compared to WT (*P* = 0.0019). **D** The mutant K562 cells were characterized by giant-multinucleated cells (20X and 100X white arrow) indicated by Hoechst staining; membrane stained with CellBrite Fix 488 (Biotium). Bottom panels are FISH on the metaphase spreads from a single WT and mutant cell using probes for Chr 3 (Orange); Chr 7 (Green); and Chr 17 (Aqua)

Giant cells have been described in various cancers and generally these giant cells are characterized by polyploidy and multiple nuclei. In order to gain insight in the ploidy of these cells we performed propidium iodide (PI) staining (Fig. 2A, B). PI-stained cells (Fig. 2C) showed a twofold increase in the number of cells with > 4 N DNA content among the mutants. This indicates that the giant cells are polyploid. Microscopic observations as well as FISH analysis revealed multiple nuclei and higher number of chromosomes in these giant cells emphasizing their polyploidy (Fig. 2D; Supplementary Figure S2 and S3).

### PGCCs in K700E mutants show better survival under stress conditions

Cellular stress is a known inducer of PGCC and frequently, PGCCs are observed after chemotherapeutic resistance [1, 4, 6, 35]. 5-Azacytidine, an FDA-approved chemotherapeutic drug for MDS patients, was used to test whether the giant cells are resistant to this agent [13]. We first determined the IC_50_ value for 5-Azacytidine for K562 cells. For this, parental K562 cells were treated with 5-Azacytidine from a low concentration of 1 µM to high concentration of 100 µM in cultured cells at time points ranging from 24 to 72 h (Supplementary Figure S4A). Cytotoxicity was determined by flow cytometry using standard Annexin V/PI staining. 5-Azacytidine at 25 µM induced apoptosis/death in 50% K562 cells in culture by 72 h (Supplementary Figure S4b). We identified this concentration as IC_50_ value for K562 and used it in subsequent experiments.

**Fig. 3:**
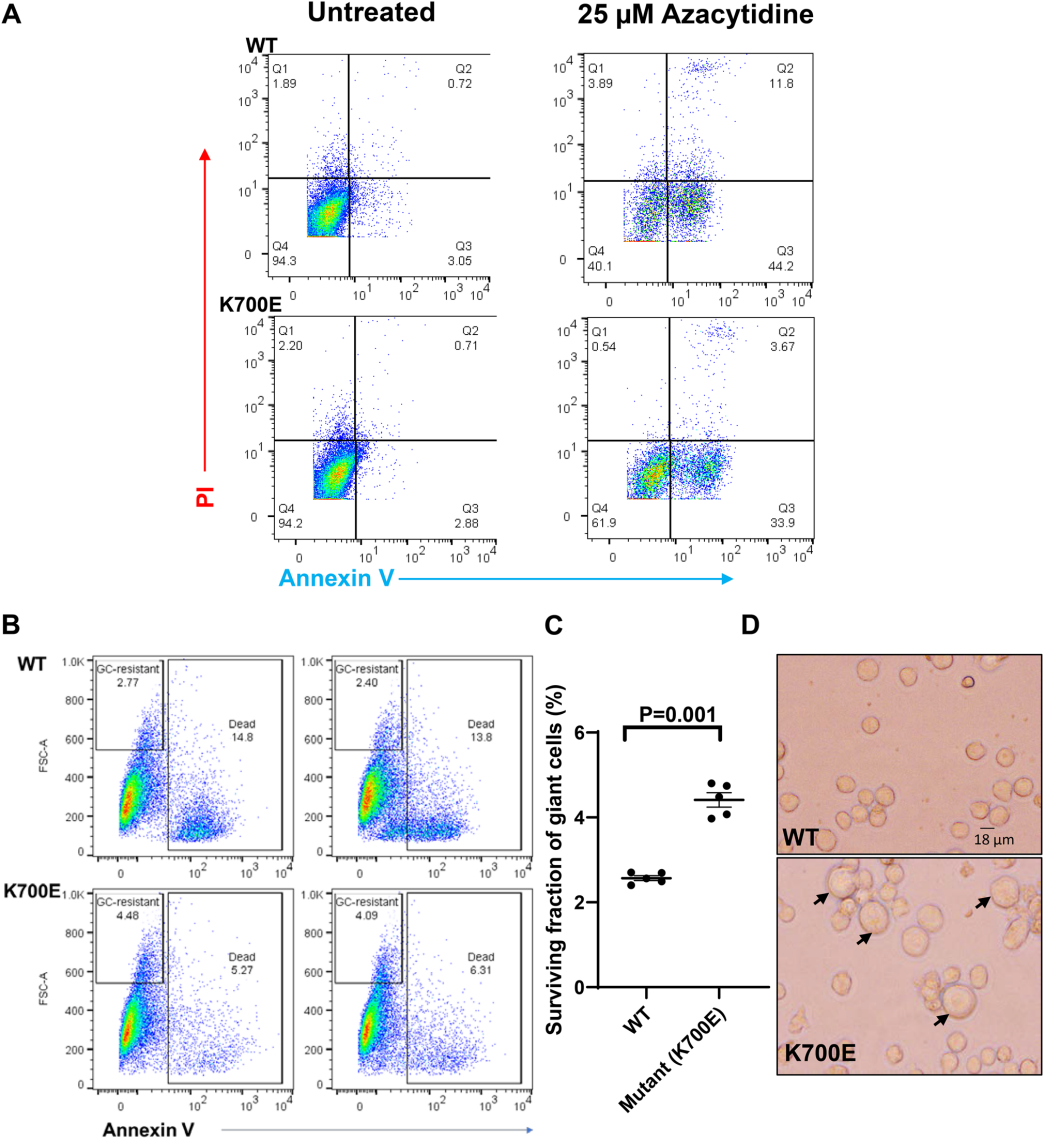
The giant cells in K700E mutants are resistant to anti-cancer drug 5-Azacytidine. **A** A representative plot showing percentage of cells that are either PI positive (Q1), Annexin V positive (Q3), Annexin V/PI double positive (Q2), and double negative (Q4) in 5-Azacytidine-treated WT and K700E mutant cells at 25 µM concentration treated for 72 h. **B** A representative flow cytometry scatter plots of five independent clones of each of WT and K700E mutant cells. Each experiment was repeated three times. Mutant cells treated with 25 µM 5-Azacytidine for 72 h show higher survival of giant cells. **C** Dot plot shows surviving fraction of giant cells between WT and K700E cells. Statistical analysis was carried out using unpaired *t* test at *P* < 0.05. **D** Microscopic images (20X) presence of giant cells in 5-Azacytidine-treated mutant K562 cells compared to WT

An equal number of WT and mutant K562 cells were treated with 25 µM 5-Azacytidine for 72 h. We observed higher cellular death in WT (55% avg.) compared to mutants K700E (31% avg.) using Annexin V/PI staining (Fig. 3A). When analyzed within the high FSC and annexin-negative fraction, the giant surviving population, we found twice as many giant cells in mutants survived the drug treatment than the WT cells (Fig. 3B, C). The same difference was observed when cells were monitored under the microscope after drug treatment with mutant K562 showing a higher number of surviving giant cells (Fig. 3D).

In order to understand the stress response in giant cells further, we cultured WT and mutant cells under serum starvation condition (Media without serum). In this condition the cells were observed at day 8 and 16 post-starvation using flow cytometry and microscopy. As expected, we saw a massive cell death in both WT and mutant cells by day 8, but the surviving fraction was relatively more in mutant. Most importantly, we observed higher surviving fraction of PGCCs in mutants compared to WT in response to starvation (Supplementary Figure S5).

### Giant cells in K700E mutants are distinct from megakaryocytes

**Fig. 4:**
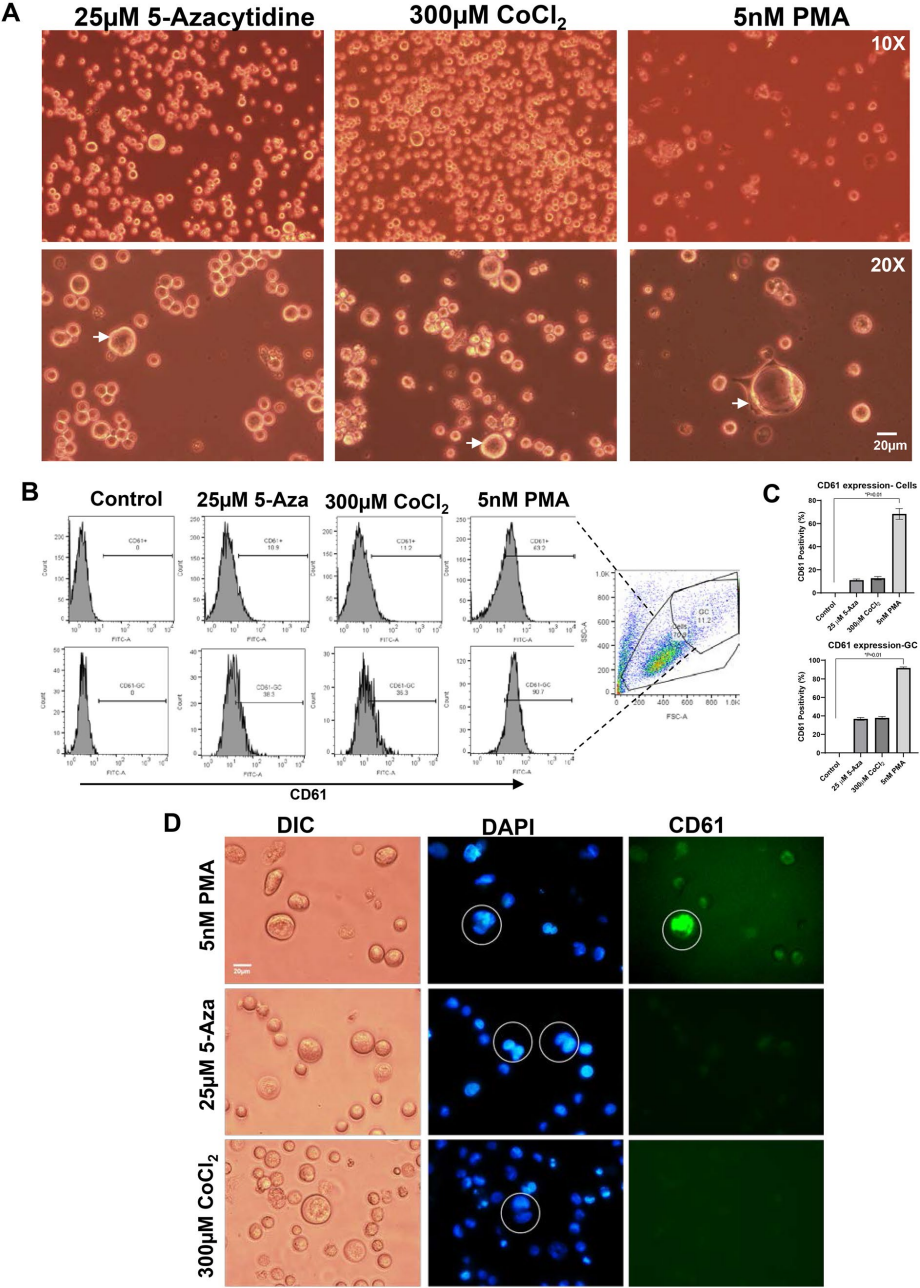
The giant cells in K700E mutants are different from Megakaryocytes. **A** K562 cells with K700E mutation were treated with 25 µM 5-Azacytidine and compared with cells treated with 5 nM PMA which have been reported to cause megakaryocyte differentiation in K562 cells. Cells treated with 300 µM CoCl_2_ were used as positive control which have been shown to cause formation of PGCCs in breast cancer cell lines. The giant cells produced by all these treatments were quite different as indicated by the representative microscopic images. **B** Representative histograms showing percentage of CD61 + mutant cells within total fraction (top row) or fraction gated as “GC” in the scatter plot showing large cells (bottom row). **C** Data from 3 independent clones are plotted in the bar diagram and analyzed using Kruskal–Wallis test with Dunn’s multiple comparisons, *Significant at *P* < 0.05. Treatment with PMA increases the expression of megakaryocyte marker CD61 in mutant cells as measured by flow cytometry. **D** Representative microscopic images showing increased expression of CD61 in PMA-treated mutant K562 cells compared to 5-Azacytidine- and CoCl_2_-treated cells

There are previous reports that K562 can be differentiated into megakaryocytes [36]. In order to determine whether giant cells produced by the mutation are distinct from megakaryocytes, we treated the WT and K700E mutant cells with PMA (Phorbol 12-myristate 13-acetate) to induce their differentiation into megakaryocytes. We compared these cells with those treated with 5-Azacytidine or CoCl_2_. CoCl_2_ has been previously reported to induce formation of PGCC by creating hypoxic condition [7]. Giant cells following different treatments appear to be morphologically distinct (Fig. 4A). PMA being a strong inducer of PKC in cells causes increased expression of adhesion molecules on the cell surface causing these cells to attach to the culture plates (Fig. 4A arrow). Analysis of well-established megakaryocyte marker CD61 on cell surface indicated a strong expression of this molecule on cells treated with PMA but not on Azacytidine or CoCl_2_-treated cells (Fig. 4B). We also found higher CD61 fluorescence in PMA-treated cells when observed under microscope (Fig. 4C). This suggests the giant cells which we observed in our previous treatments are not megakaryocytes.

### Giant cells are proliferative and produce normal-sized progeny

**Fig. 5:**
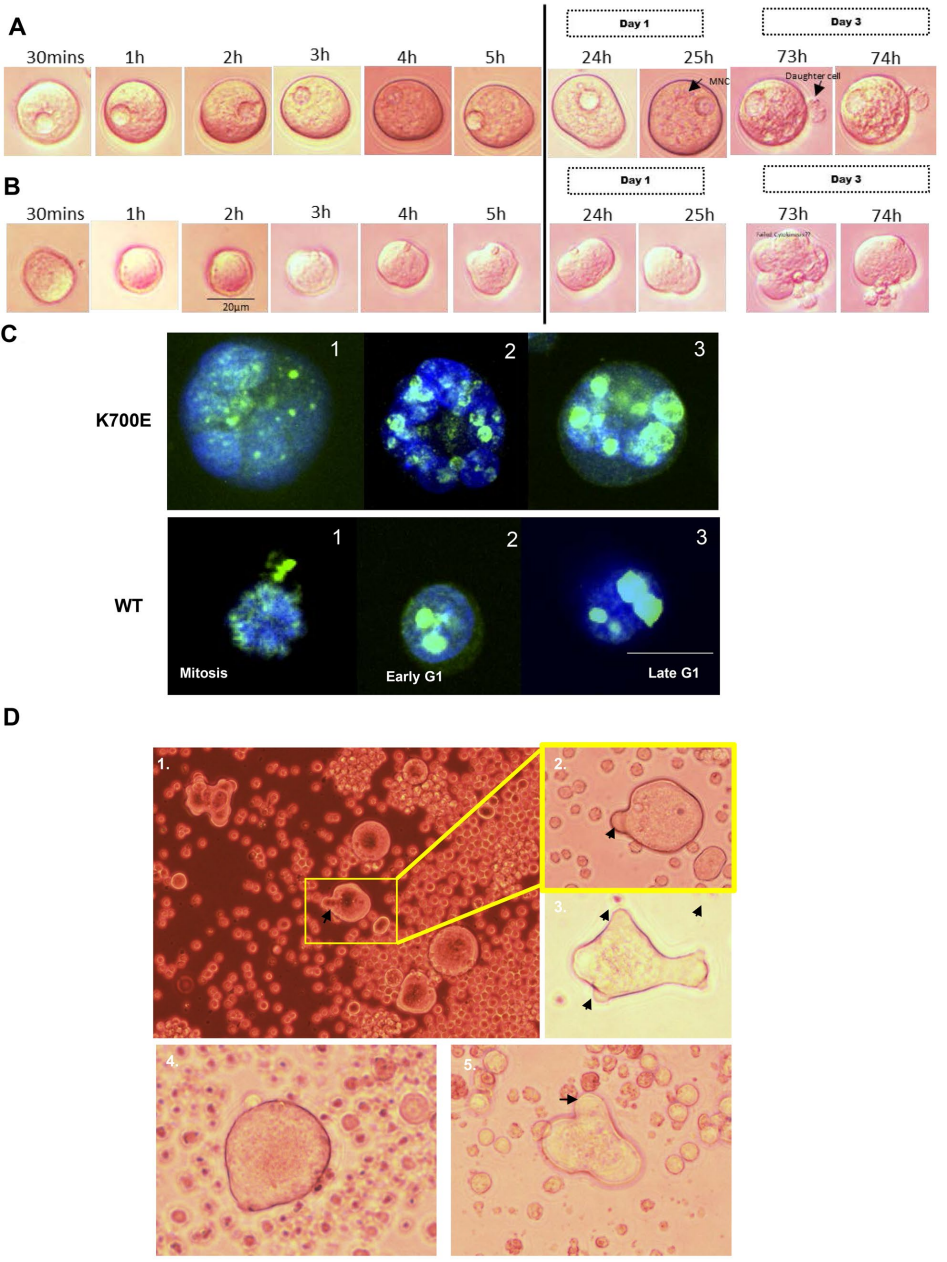
The giant cells produce small-sized progeny cells. K562 cells with K700E mutation were seeded in 96-well plate at a density of 100 cells per well. Out of them one giant cell/well (1 and 2) was followed for 3 days and images were taken every hour. The images with any event are shown in the figure. Isolated giant cells produce single (**A**) or multiple (**B**) progeny cells of smaller size by 73-h post-seeding. **C**. Ki-67 staining in WT and K700E cells at different stages of cell cycle indicating they are dividing. Images were captured at 40X using confocal microscope. Bar indicates 15 µm. In culture the giant cells are capable of cell fusion. **D**. K562 cells with K700E mutation develop finger-like projections (black arrows in 1–5) to bind and fuse with neighboring small-sized cells (Time-lapse video in Supplementary Figure S7)

It is reported that PGCCs contribute to relapse in cancers following chemotherapy by producing normal-sized diploid cells [7]. In order to see whether these giant cells proliferate in culture, we monitored them through time-lapse microscopy. Mutant giant cells were seeded in 96-well plate at a very low concentration with methylcellulose-containing media (Stem Cell Methocult) to restrict their movement. Cells were followed for 24 to 96 h by keeping them in CO_2_ incubator and imaging them every 1 h. For initial 48 h (Fig. 5A), we did not observe any cell division in these cells but by 73-h post-seeding we found single or multiple small-sized daughter cells in the periphery of the giant cells (Fig. 5B). These daughter cells divide rapidly to overwhelm the entire culture. The daughter cells were observed to be produced through the process of budding (Supplementary Figure S6). We stained these cells for Ki-67, a proliferation marker, and show that the giant cells are actively dividing cells (Fig. 5C).

### Giant cells are capable of cell fusion

We monitored these giant cells in culture through time-lapse microscopy making sure each giant cell was followed for 24 h and imaged every 30 min. Mutant K562 cells were grown in Stem Cell MethoCult media at a concentration of 10^4^ cells per plate, incubated in Biospa, and imaged using BioTek Microscope. Mutant cells produced finger-like projections to bind and fuse with neighboring cells to become larger in size (Fig. 5D). The formation of finger-like projections was also observed in cultures after treatment with the anti-cancer drug (Fig. 5D). A composite video of time-lapse microscopy revealed cell fusion in real time (Supplementary File S7).

### PGCCs in mutant K562 cells have higher accumulation of mitochondria

**Fig. 6:**
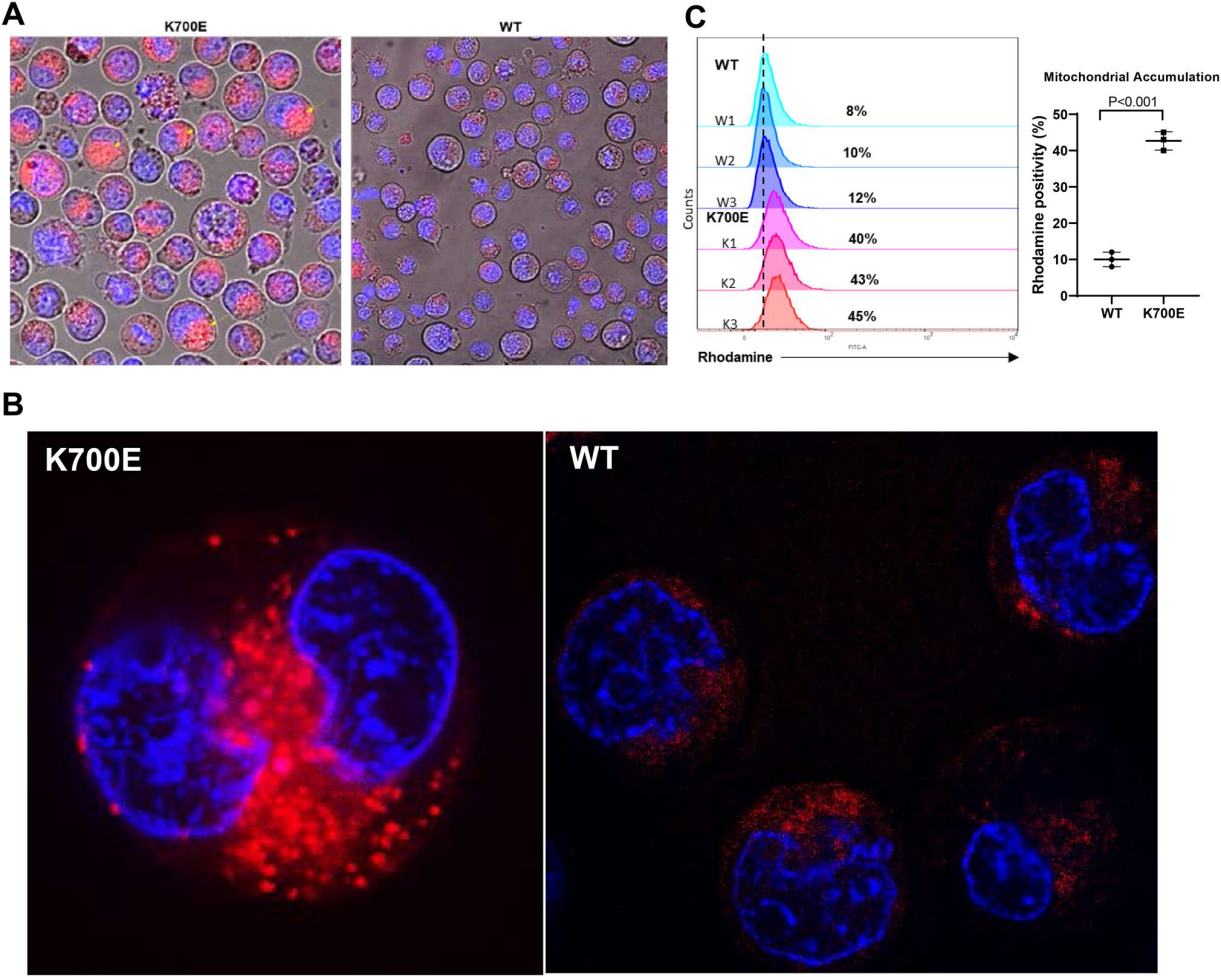
The giant mutant (K700E) K562 cells have higher accumulation of mitochondria. K562 cells with K700E mutation were stained with Hoechst (Thermo) for nuclear and Rhodamine 123 (Thermo) for mitochondrial visualization. Cells were seeded in 35-mm glass bottom dishes (MatTek) at a density of 1000 cells/well and imaged in Nikon A1 Confocal Microscope. **A** Representative microscopic images showing higher accumulation of mitochondria in mutant (K700E) compared to WT K562 cells. **B** Enlarged images of two individual giant cells in mutant K562 cells showing distribution of mitochondria inside the cells. **C** A representative histogram of three independent experiments each showing mitochondrial biomass in three independent clones measured using Rhodamine staining-based Flow cytometry. Mutants (K700E) showed on an average 4 times more rhodamine fluorescence than equal number of WT cells that was statistically significantly different (unpaired *T*-test; *P* < 0.001)

In MDS, mutations in *SF3B1* gene are commonly associated with ring sideroblasts that owe their peculiar appearance to iron-filled mitochondria. We investigated the biodistribution of mitochondria by staining WT and mutant K562 cells with a mitotracker Rhodamine 123, a fluorescent dye. Fluorescent intensity was measured using flow cytometry and visual observations were made using confocal microscopy. A significantly higher amount of Rhodamine accumulated in K700E mutant K562 compared to WT cells (Fig. 6). Giant cells had higher mitotracker accumulation than normal-sized K562 cells, presumably due to larger size.

## Discussion

PGCCs are rare cells that are observed both in vivo (biopsies from patients) and in vitro (primary cell cultures and cell lines) in studies dating back to over two centuries, but their significance has been appreciated more in the last decade as functional studies mainly in solid tumors implicated their role in tumorigenesis, metastasis, and therapy resistance [1–4, 6, 8, 12, 37–39]. Multinucleated giant Reed–Stenberg cells [40] are a hallmark of Hodgkin’s lymphoma, but evidence of PGCCs in liquid cancers is sparse consisting of case reports of tetraploid cells with giant cell morphology [18–21]. Monocyte-derived macrophages were shown to form multinucleated giant cells and act as nurse cells that de novo generate other cell types in vitro [41]. Also, multinucleated giant cells are observed around new bone material after implantation [42]. Taken together, it appears that multinucleated giant cells are formed in response to stress and play important roles in a variety of human diseases, including cancer. The exact origin of these cells is not clear, but evidence, in a few cases, supports the role of monocyte macrophages that fuse to generate these cells. Finally, the genes and pathways involved in the formation of multinucleated giant cells are not known.

In our attempt to study the molecular and cellular defects due to *SF3B1* mutation, we observed that introduction of the hotspot mutation in K562 cells, a continuously growing cell line derived from chronic myelogenous leukemia in terminal blast crises widely used as a model for myeloid malignancies, resulted in an increased frequency of giant cells in multiple clones derived from mutant cells but not in wild-type clones. The presence of increased number of giant cells in mutants was confirmed both by microscopic observations and flow cytometry using forward (FSC) and side (SSC) scatter. Typically, cells with large size have increased FSC. The increase in giant cells in the mutant, and not wild-type, is unrelated to the CRISPR/Cas9-mediated genomic alterations since the wild-type cells were similarly manipulated by introducing a synonymous change (Fig. 1A) next to the mutant nucleotides. Also, the giant cell formation was found to be specific to K700E mutation in *SF3B1* gene as introduction of another well-established mutation (P95H) in *SRSF2* gene did not result in increased giant cell formation in K562 cells (Supplementary Figure S1). It is interesting to note that macrocytic anemia which is characterized by giant cells is frequently present in MDS subtype with increased ring sideroblasts and thrombocytosis with SF3B1 mutations [25, 43]. Also, mice or zebra fish carrying SF3B1 K700E mutations show macrocytic anemia suggesting this mutation is associated with increase cell size in certain cell types. Since PGCCs are characterized by polyploidy and multiple nuclei, DNA content was calculated using several independent measures that include propidium iodide staining, metaphase and FISH analysis, and Hoechst staining followed by microscopy. In multiple independent clones, PI staining showed that giant cells were polyploid with > 6 N DNA content (K562 is a near triploid). This polyploidal nature of giant cells was confirmed using FISH analyses on metaphases, where greater than 3 N chromosomes and multiple copies of FISH probes asymmetrically distributed among multiple nuclei were documented. Interestingly, FISH analysis also found evidence of “mitotic catastrophe,” which, in the context of giant cells, is believed to be due to abnormal or incomplete mitoses frequently observed in PGCC, resulting in aneuploid or nearly diploid daughter cells [44–49].

PGCCs in solid tumors are observed after radiation or chemotherapeutic interventions and have been well documented both in patient biopsies and in vitro studies [3, 5, 8, 9, 11, 35, 38, 50–52]. PGCC formation is believed to be a natural response to stress [4, 5, 53]. Azacytidine, a DNA methylation inhibitor, is approved for use in MDS and AML [54]. Similar to PGCCs in solid tumors [53], we found PGCC in this liquid cancer to be resistant to Azacytidine at a dose that killed half the K562 cells as evidenced by an enrichment of PGCCs. In response to Azacytidine, we showed a higher percentage of death in WT cells and a higher percentage of surviving fraction of giant cells in mutants (K700E) indicating these cells are resistant to the anti-cancer drug (Fig. 3). We observed similar resistance of giant cells to stress induced by serum starvation. Short-term serum starvation results in cell cycle arrest and is commonly used in cell synchronization methods, but long-term starvation results in cellular apoptosis and death. The giant cells persisted after 18 days of starvation with mutants showing higher survival in culture compared to WT cells (Supplementary Figure S5).

One concern regarding the use of K562 cells is that they can differentiate toward erythroid or megakaryocyte lineages under appropriate conditions [55]. Specifically, hemin treatment results in increased differentiation of these cells to erythrocyte lineage [56] and PMA treatment to megakaryocyte lineage [36]. To rule out that these giant cells are not spontaneously differentiated megakaryocyte, we stained the cells with CD61, a marker for megakaryocyte differentiation (Fig. 4B and C). We also stained cells that were treated with either azacytidine or CoCl_2_ that is known to create hypoxic conditions and increase PGCC formation [7] and PMA that is well established to differentiate K562 into mature megakaryocytes. Unlike PMA treatment, where there was a robust increase in CD61 uniformly in all cells, we noticed a modest increase in CD61 expression with Azacytidine or CoCl_2_ treatment, likely due to deregulation of gene expression (Fig. 4B). Most importantly, the giant cell morphology and nuclear morphology (which is typically multilobulated in megakaryocytes) observed in PMA-treated cells are distinct from that observed with 5-Azacytidine or CoCl_2_ treatment suggesting the PGCCs are not megakaryocytes but a distinct subset of cells (Fig. 4A and C).

The multinucleated state of PGCCs is achieved by cell fusion and/or nuclear division with failed cytokinesis [5]. Also, PGCCs are known to divide giving birth to normal-sized cells with near normal ploidy by asymmetrically dividing their genome content [44, 48]. Time-lapse microscopy combined with Ki-67 proliferation marker data suggested that the giant cells observed in mutants are capable of cell division to give rise to daughter cells (Fig. 5). Additionally, we observed various morphological states of giant cells in culture with appearance of budding or bursting as documented in PGCCs from solid tumors (Supplementary Figure S6). We also observed cell fusion happening in culture when giant cells are in the vicinity of smaller cells (Supplementary File S7). Our observations indicate that these giant cells are capable of both endoreduplication as well as cell fusion and can give rise to small size progeny as reported previously for solid tumors [5].

As noted earlier, mutations in *SF3B1* gene are associated with the appearance of ring sideroblasts; erythroblasts characterized by iron laden mitochondria surrounding the nuclei of erythroblasts. We observed a 3–4fold increase in mitochondrial biomass in mutant cells compared to wild-type but the significance of this observation in the formation and functions of PGCC is not clear (Fig. 6).

The significance of our observations is that we document PGCCs in a cell line derived from a hematological cancer for the first time. We show that the increased frequency of PGCC formation is linked to a specific genetic event, that is, introduction of a point mutation in a splicing factor, suggesting possible distinct molecular mechanisms for PGCC formation.

## Supplementary Information

Below is the link to the electronic supplementary material.

Supplementary file1 (PDF 1072 kb)

Supplementary file2 (AVI 87 kb)

## Data Availability

The datasets generated during and/or analyzed during the current study are available from the corresponding author on reasonable request.
