# Supplementary material for: Mutation in SF3B1 gene promotes formation of polyploid giant cells in Leukemia cells
Source: Med Oncol. 2022 Apr 28;39(6):65. doi: 10.1007/s12032-022-01652-9 (PMC9046281; doi:10.1007/s12032-022-01652-9)
Supplement: Supplementary file 1 — Supplementary file1 (PDF 1072 kb) [file 12032_2022_1652_MOESM1_ESM.pdf]

Mutation in *SF3B1* gene promotes formation of polyploid giant cells in Leukemia cells

Sanjay Mukherjee, PhD, Abdullah Mahmood Ali, PhD, Vundavalli

V Murty, PhD, and Azra Raza, MD

Mutation in *SRSF2* gene does not induces Giant Cell Formation above basal levels

a.

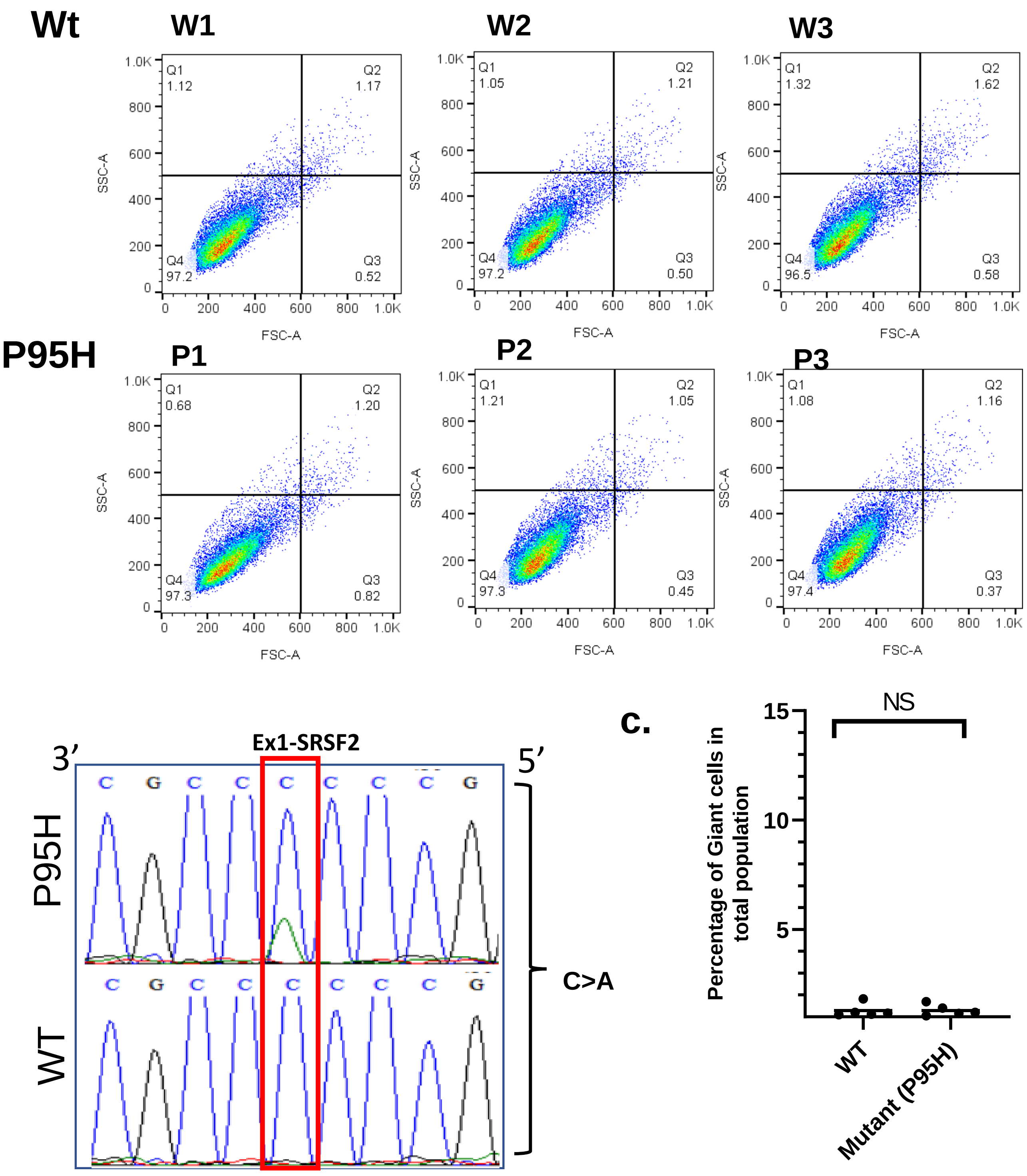

Supplementary Figure S1. **a.** Mutation(P95H) in SRSF2 gene doesn't induces Giant Cell Formation. K562 cells were engineered using CRISPR/Cas9 method to introduce c.284C>A (Substitution, position 284, C➞A) **b**. Sequence chromatogram showing the location of mutation (Panel **b** is in reverse complement) which results in addition of Histidine (H) in place of Proline (P) at 95 amino acid position in SRSF2 protein. **c.** There was no difference in the percentage of giant cells based on Flow cytometry (FSC vs SSC) between WT and mutant clones (Statistical analysis using Unpaired T-test [Welch's correction],n=3).

Giant Cells in Mutants(K700E) have higher genome (>2N)

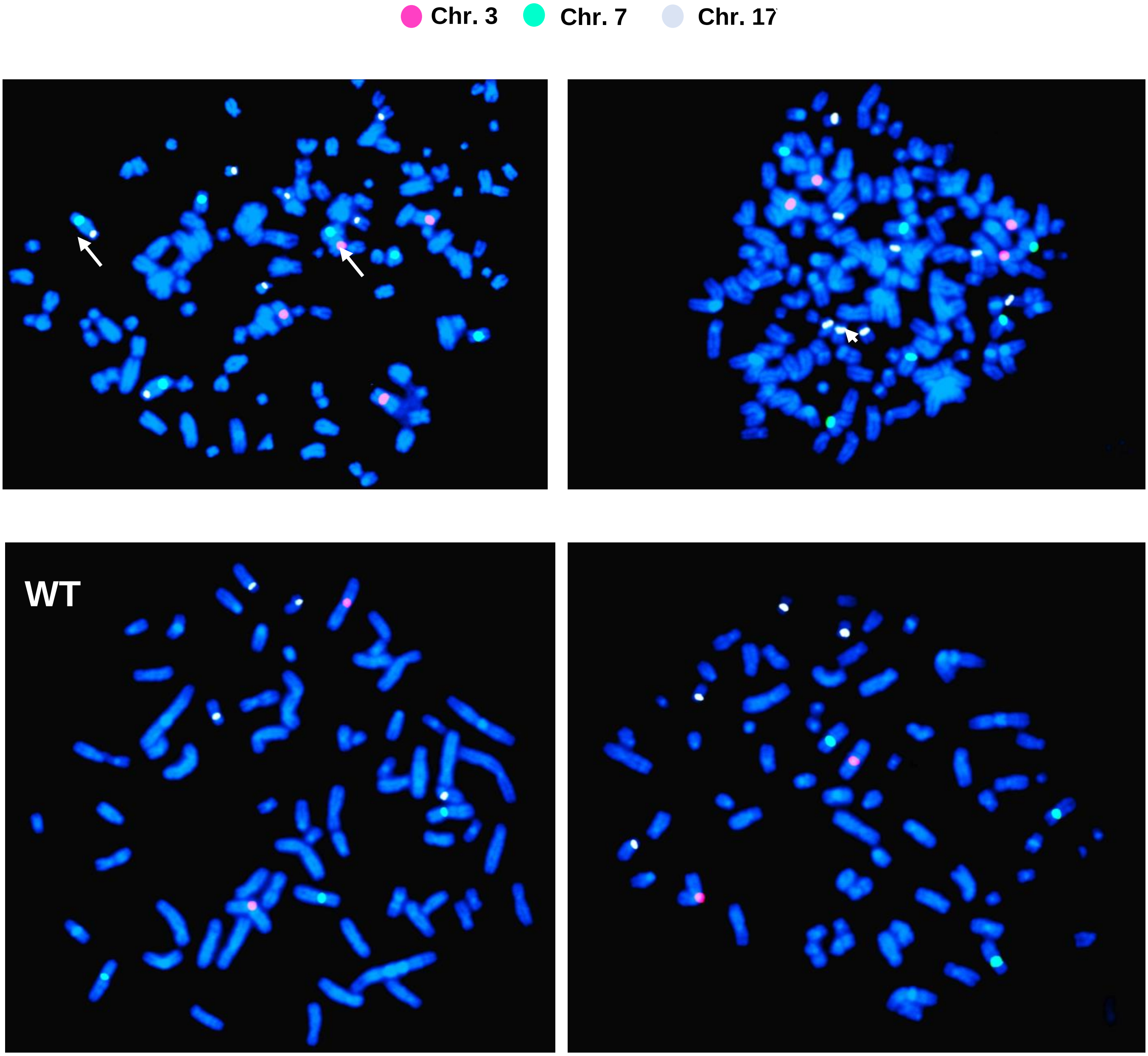

Supplementary Figure S2. FISH on metaphase cells of Giant Cells in Mutants (K700E) show polyploid (>4N) chromosome content and show increased chromosomal abnormalities (white arrows).

Giant Cells have Multiple nuclei with abnormal chromosomal distribution

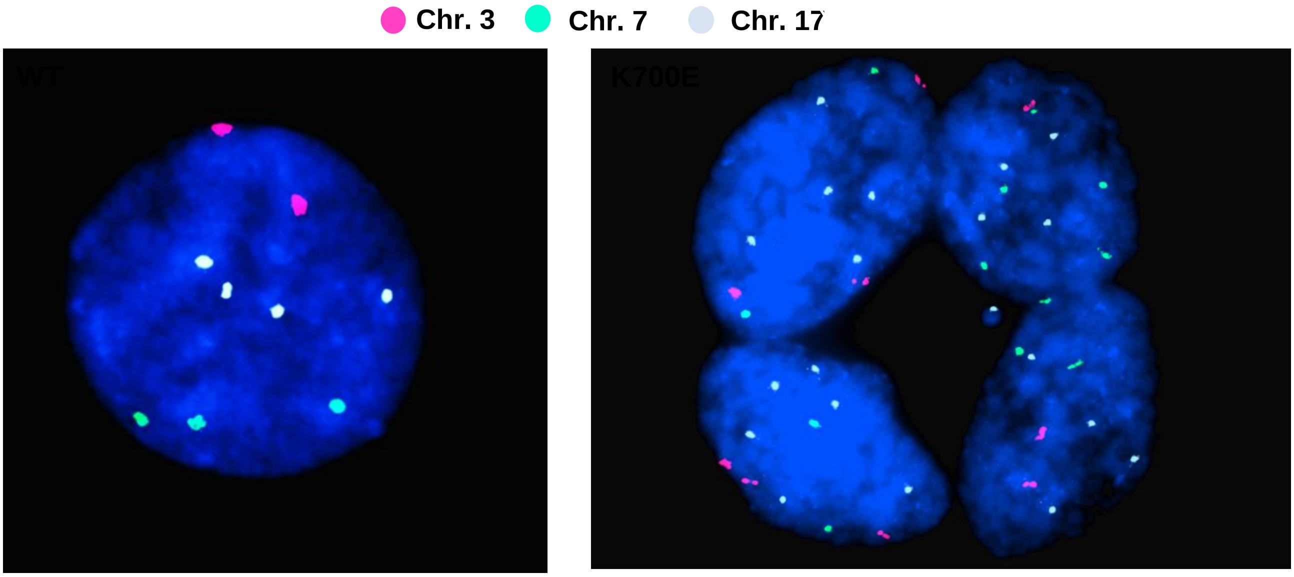

Supplementary Figure S3. Interphase FISH analysis of Giant Cells in mutants (K700E) have multiple nuclei with abnormal chromosomal distribution (right panel) compared to WT cells (left panel).

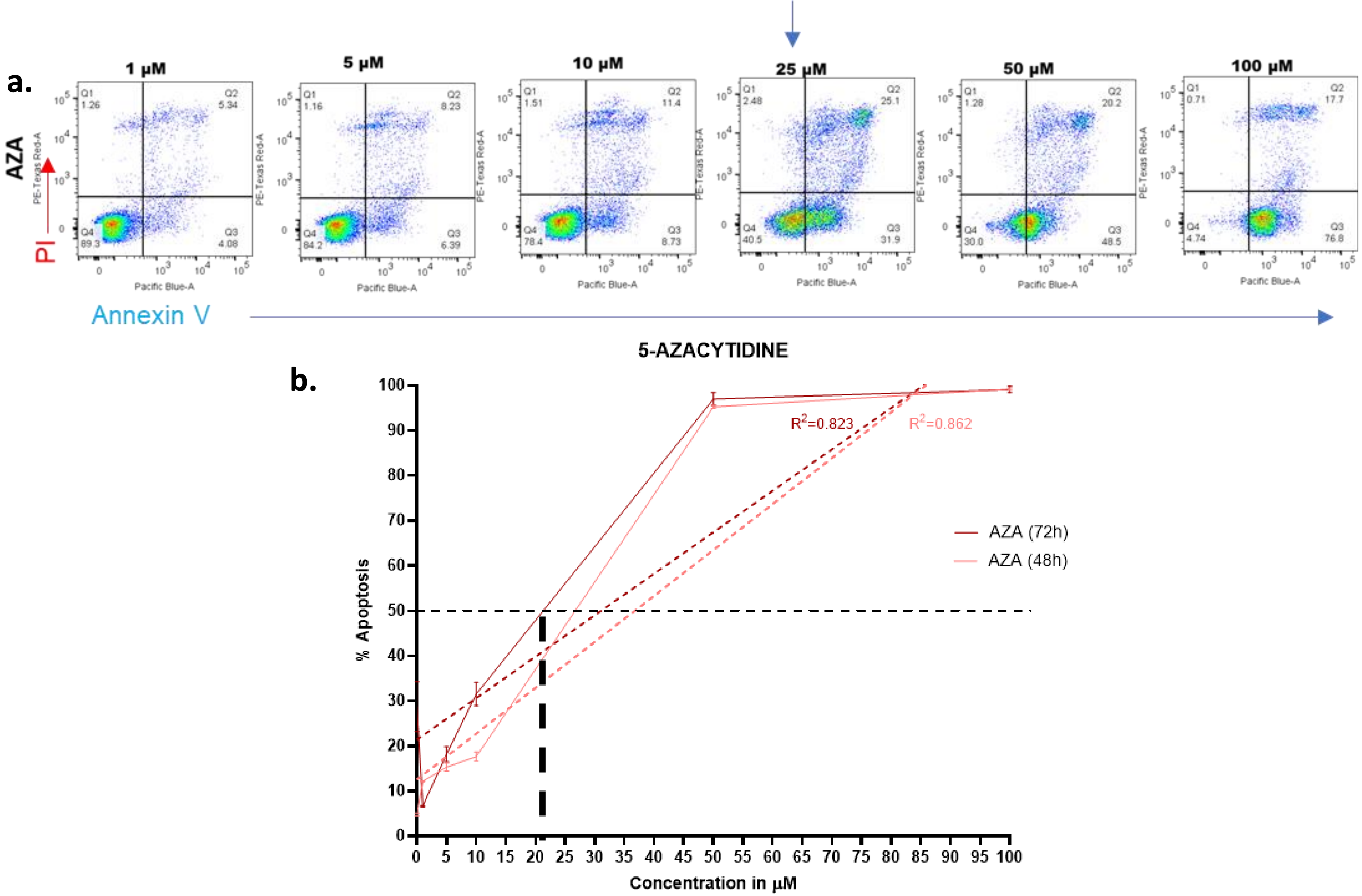

Supplementary Figure S4. Effect of 5-Azacytidine in K562 cells **a**. Cytotoxicity assay (Annexin V/PI) for 5-Azacytidine in K562 cells at different concentration**. b**. 5-Azacytidine kills 50% of K562-cells by 72h at a concentration of 25µM. The data is represented as mean±SD of 3 independent experiments at 2 time points (48h,72h).

Supplementary Figure S5

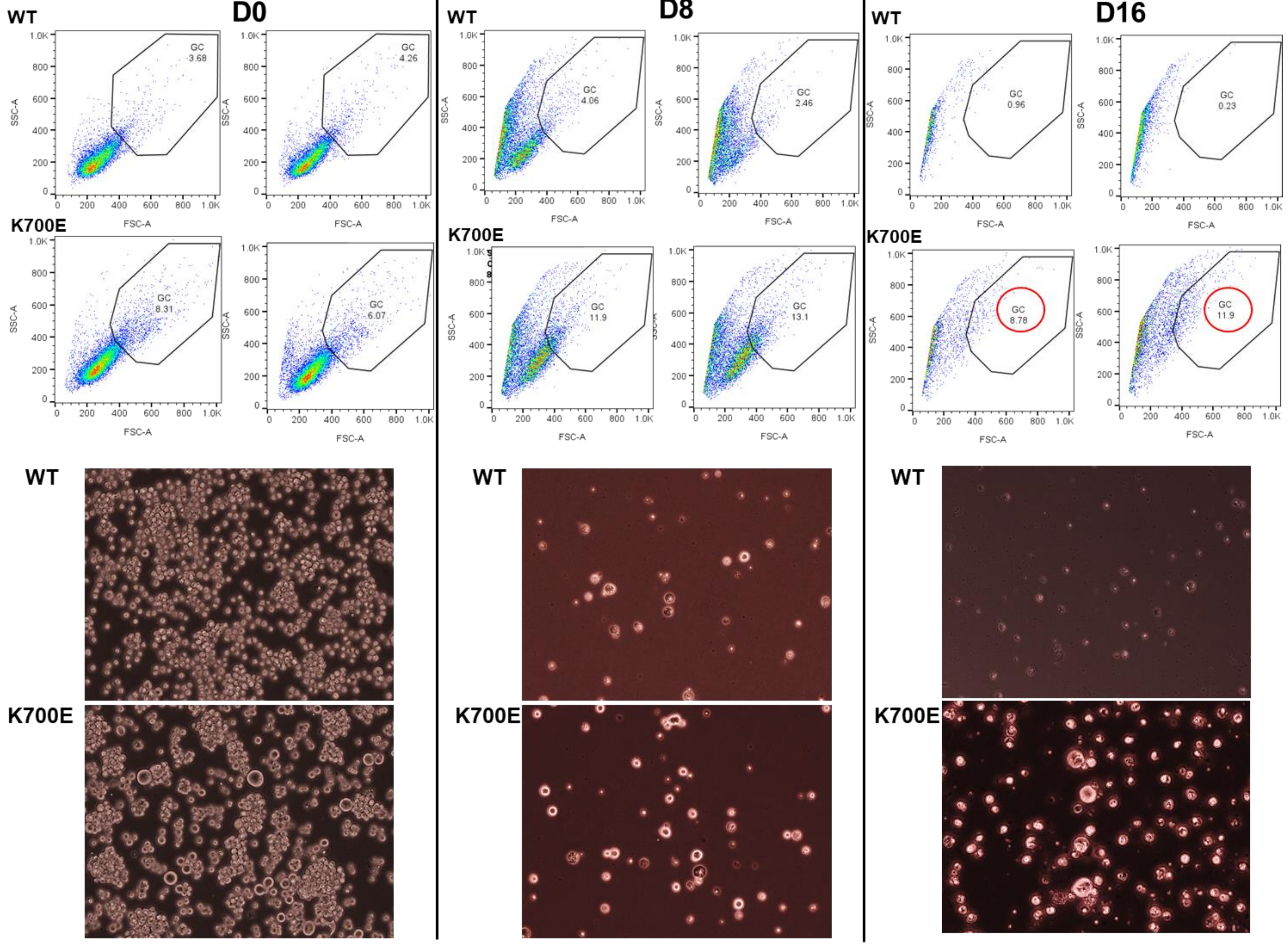

Supplementary Figure S5. Effect of serum starvation on giant cells in WT and mutant cells. Cells were cultured in serum free media for 16 days and analyzed using A. flow cytometry (FSC vs SSC) and B. Microscopy.

The giant cells give rise to small cells through budding.

a. A single giant cell giving rise to multiple small cells. A specific microscopic field(yellow box) has been shown at 3 different magnifications (10X,20X, 40X)

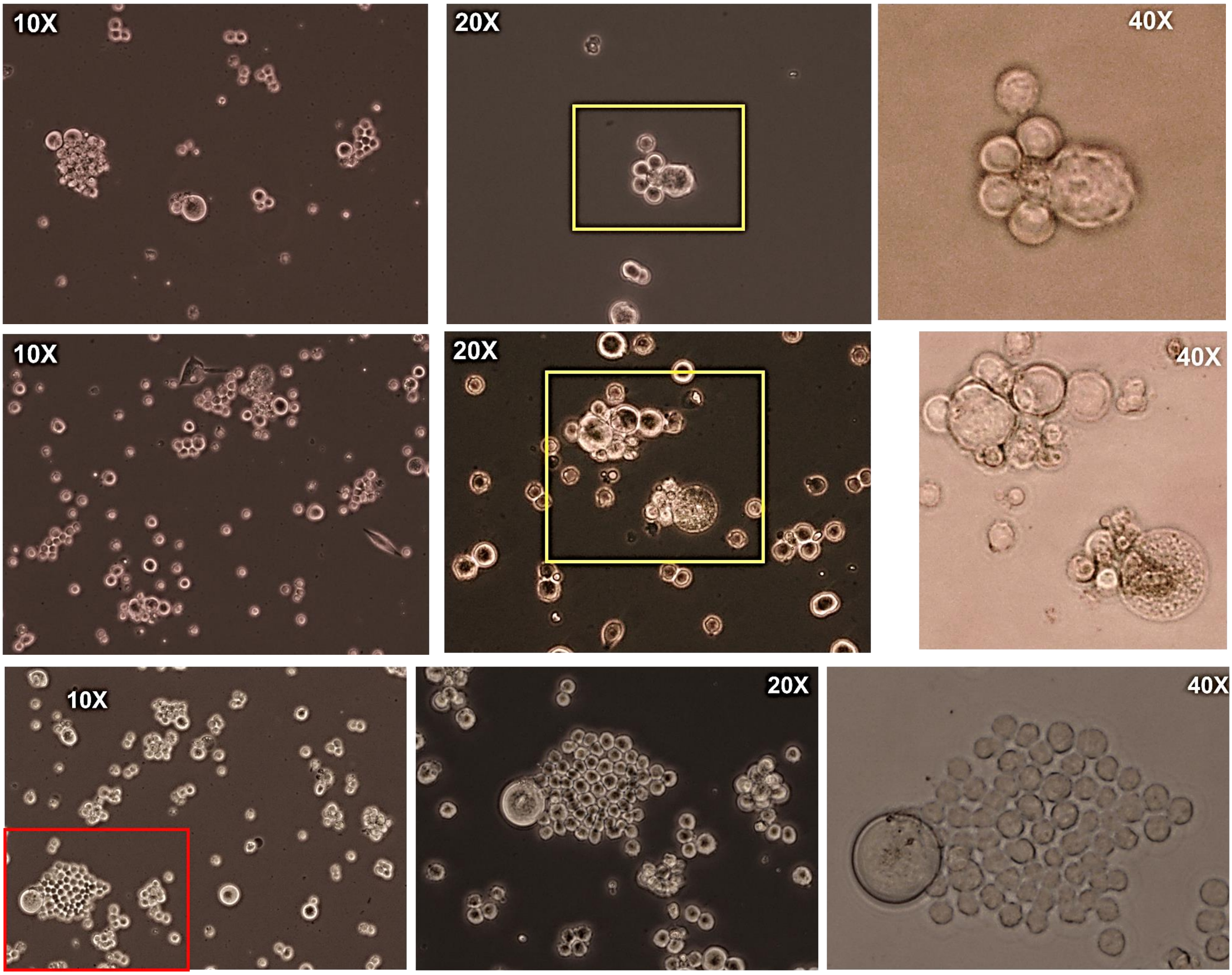

b. The budding process(red arrow) from a giant cell has been captured at higher magnification (40X)

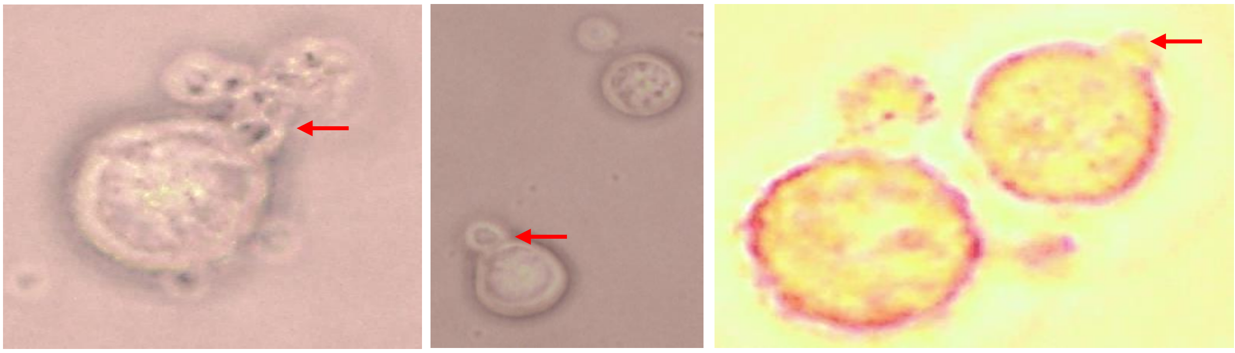

Supplementary Figure S6. The giant cells give rise to small cells through budding. **a**. A single giant cell giving rise to multiple small cells. A specific microscopic field (yellow box) has been shown at 3 different magnifications (10X,20X, 40X) **b**. The budding process (red arrow) from a giant cell has been captured at higher magnification (40X)
